# Gut microbiome-driven mechanisms in inflammatory bowel disease-associated interstitial lung disease: from dysbiosis to therapeutic implications

**DOI:** 10.3389/fcimb.2026.1967125

**Published:** 2026-09-23

**Authors:** Yanli Yang, Dongmei Zhang, Wenwen Liu

**Affiliations:** Respiratory Department, First Hospital of Jilin University, Changchun, China

**Keywords:** bacterial translocation, gut-lung axis, inflammatory bowel disease, interstitial lung disease, microbial metabolites, microbiome, multi-omics

## Abstract

Inflammatory bowel disease (IBD) is a chronic, relapsing immune-mediated disorder of the gastrointestinal tract. Interstitial lung disease (ILD) associated with inflammatory bowel disease has been receiving increasing attention. With advances in the gut-lung axis theory, accumulating epidemiological and mechanistic evidence indicates that the association between IBD and ILD is not coincidental or merely driven by drug toxicity, but rather reflects a systemic disease spectrum involving microbiome dysbiosis and cross-organ immune dialogue. This article presents a critical narrative review that systematically summarizes the mechanisms underlying IBD-related ILD, focusing on causal evidence from large cohort and Mendelian randomization studies. It also looks at how gut microbial metabolites remotely regulate lung immune homeostasis, the inflammatory cascades triggered by gut barrier disruption and bacterial translocation, and the therapeutic potential of microbiome-based interventions. This article examines the main limitations and controversies, including the limited accuracy of animal models, the unclear causal link between microbial changes and disease onset, and the challenges in selecting clinical trial endpoints. It also outlines future research directions, focusing on integrating multi-omics, non-invasive diagnostic models, and strategies to distinguish drug-induced lung injury from primary disease involvement. This review aims to elucidate the pathogenesis of this cross-organ disease spectrum and to provide a theoretical foundation for both basic research and clinical translation.

## 1 Introduction

Inflammatory bowel disease (IBD) is a complex group of immune-mediated disorders characterized by chronic, spontaneous, and recurrent inflammation of the gastrointestinal tract (Bueno et al., 2022; Zhong et al., 2023; Fu et al., 2025). The pathogenesis of IBD has not yet been fully elucidated, but it is generally believed to result from a dysregulated immune response in genetically susceptible individuals to environmental factors and the gut microbiota, manifesting as excessive secretion of pro-inflammatory cytokines and disruption of the intestinal barrier function (Agrawal et al., 2022; Esposito et al., 2022; Lee et al., 2024). IBD is primarily classified into two types. Crohn’s disease (CD) is characterized by inflammation that can affect the entire gastrointestinal tract, from the mouth to the anus (Pagani et al., 2022). Ulcerative colitis (UC), on the other hand, primarily causes inflammation and ulcers in the colon and rectum, with lesions confined to the distal gastrointestinal tract (Pagani et al., 2022).IBD is characterized by chronic, recurrent intestinal inflammation, but its systemic nature has long been recognized clinically through extraintestinal manifestations across multiple organs (Pagani et al., 2022). IBD-related pulmonary involvement is considered a rare extraintestinal manifestation. Combined with its insidious onset and tendency to be confused with drug toxicity or opportunistic infections, its clinical significance is often severely underestimated (Taelman et al., 2023; Mu et al., 2026).

Epidemiological studies in recent years have shown that 40% to 60% of IBD patients have subclinical pulmonary interstitial infiltrates or abnormal lung function, and that pulmonary lesions can affect any part of the respiratory system (Moeser et al., 2018). Furthermore, retrospective cohort studies indicate that IBD patients have a significantly increased risk of developing bronchiectasis, chronic bronchitis, and asthma, and this association is more consistent and pronounced in CD than in UC (Ailawadi et al., 2026; Ding et al., 2026). As the theory of the “gut-lung axis” has evolved, the research focus has shifted from the pathological description of individual organs to exploring microbiome interactions and mechanisms of systemic immune dialogue between the two (Druszczynska et al., 2024; Liu et al., 2026). In this critical narrative review, we aim to systematically summarize the molecular mechanisms of dialogue underlying IBD complicated by interstitial lung disease. By examining everything from microbiome dysbiosis to metabolite-mediated immune regulation and bacterial translocation, this review elucidates the pathogenesis of this cross-organ disease spectrum and provides a theoretical basis for future precision diagnosis and targeted therapy.

## 2 Epidemiological associations between IBD and interstitial lung disease

Large-scale population-based cohort studies and Mendelian randomization analyses have provided robust epidemiological evidence linking IBD to ILD (**Table 1**). The Swedish National Cohort Study (85,705 IBD patients) reported an ILD incidence rate of 34 per 100,000 person-years in IBD patients, versus 20 per 100,000 person-years in the general population (HR = 1.48, 95% CI 1.30–1.69); the risk remained elevated even when compared with unaffected siblings (HR = 1.81) (Kochar et al., 2026). Similarly, a 45-year nationwide cohort study in Denmark also showed that the cumulative incidence of ILD among IBD patients was 2.1%, approximately twice that of the control group (0.9%), with an adjusted HR of 2.1 (95% CI 1.8–2.4) (Baekdal et al., 2026). A US retrospective study of 8,865 IBD patients reported a 4.6% concurrent ILD prevalence, with significantly higher burden in UC than CD (P < 0.001) (Parekh et al., 2026). A case-control study enrolled 114 patients with inflammatory bowel disease and 120 controls. The results showed that vital capacity (VC), forced vital capacity (FVC), forced expiratory volume in one second (FEV1), peak expiratory flow (PEF), and maximal mid-expiratory flow (MMEF) were all significantly reduced in patients with inflammatory bowel disease (Zhao et al., 2017).In a 3-year pediatric study (100 IBD vs. 50 controls), FEV1, FVC, FEF_25–75_% (forced expiratory flow at 25% to 75% of vital capacity), and diffusing capacity of the lung for carbon monoxide (DLCO) showed progressive declines (P < 0.05), worsened during active disease, correlated negatively with activity scores, and persisted in remission, confirming early subclinical lung involvement (El Amrousy et al., 2018). At the genetic level, a two-sample Mendelian randomization study confirmed a causal association between genetic susceptibility to IBD and increased ILD risk (OR = 1.20, 95% CI 1.17–1.24), with both CD and UC showing significant effects (Luo et al., 2023).In addition to an increased risk of ILD, patients with IBD also experience a significant burden of respiratory symptoms. A French prospective cross-sectional study of 325 IBD patients showed that 50.8% had respiratory symptoms, among whom 42.2% of those referred to pulmonology were confirmed with lung diseases (Valentin et al., 2023).In addition, a nationwide retrospective cohort analysis in the United States showed that the age-adjusted mortality rate due to ILD among IBD patients exhibited a significant upward trend between 2001 and 2020, and the proportion of deaths attributable to ILD also increased year by year (Vaezi et al., 2024).In summary, studies from various countries employing diverse designs have consistently confirmed a robust epidemiological association between IBD and ILD, which may be causal, suggesting that clinical practice should emphasize monitoring pulmonary manifestations and long-term respiratory health in patients with IBD.

**Table 1:** Summary of major epidemiological studies on the association between inflammatory bowel disease and interstitial lung disease.

Study	Design	Sample size	Key finding	Statistical significance/remarks
Swedish nationwide cohort study (Kochar et al., 2026)	Population-based prospective cohort	85,705 IBD patients vs. general population & unaffected siblings	ILD incidence: 34 vs. 20 per 100,000 person-years; HR = 1.48 (1.30–1.69); sibling-controlled HR = 1.81	Significantly elevated risk; suggests genetic and environmental contributions
Danish nationwide 45-year cohort study (Baekdal et al., 2026)	Population-based retrospective cohort	National IBD cohort vs. matched controls	Cumulative ILD incidence: IBD 2.1% vs. control 0.9%; adjusted HR = 2.1 (1.8–2.4)	Approximately twofold increased risk; highly significant
US multi-center retrospective study (Parekh et al., 2026)	Retrospective cross-sectional/case series	8,865 IBD patients	Concurrent ILD prevalence 4.6%; significantly higher in UC than CD (P < 0.001)	Suggests UC may have a stronger association with ILD
Case-control study (China) (Zhao et al., 2017)	Cross-sectional case-control	114 IBD vs. 120 healthy controls	VC, FVC, FEV1, PEF, MMEF significantly decreased in IBD (P < 0.01); no correlation with disease extent/severity; elevated ESR identified as risk factor	Highlights subclinical physiological impairment; supports proactive PFT screening in high-ESR patients
3-year pediatric observational study (Egypt) (El Amrousy et al., 2018)	Longitudinal case-control	100 newly diagnosed IBD (52 CD, 48 UC) vs. 50 matched controls	Progressive declines in FEV1, FVC, FEF_25–75_%, DLCO over 3 years (P < 0.05); worse during active disease; negatively correlated with activity scores; abnormalities persisted in remission	Confirms early-onset, progressive subclinical impairment; supports routine PFT monitoring in pediatric IBD even when asymptomatic
French prospective cross-sectional study (Valentin et al., 2023)	Prospective cross-sectional survey	325 IBD patients	50.8% had respiratory symptoms; 42.2% of those referred to pulmonology had confirmed lung disease	Highlights high prevalence of subclinical pulmonary involvement
US national mortality trend analysis (Vaezi et al., 2024)	Population-based retrospective trend analysis	IBD death data from 2001–2020	Age-adjusted ILD-attributable mortality showed a significant upward trend (P < 0.05)	ILD is an increasingly important cause of death in IBD patients
Two-sample Mendelian randomization study (Luo et al., 2023)	Genetic causal inference (MR)	Genome-wide association study (GWAS)summary data for IBD and ILD	Genetically predicted IBD → ILD: OR = 1.20 (1.17–1.24); both CD and UC significant	Supports causality, independent of confounding

## 3 Mechanisms of microbial regulation mediated by the gut-lung axis

The gut-lung axis functions as a complex bidirectional communication network, primarily via the microbial community and its metabolites, which regulate immune homeostasis remotely (He et al., 2017; Tan et al., 2020). In IBD-ILD pathophysiology, gut microbiota dysbiosis serves not only as a hallmark of local intestinal lesions but also as a key systemic driver of lung injury (**Figure 1**).

**Figure 1:**
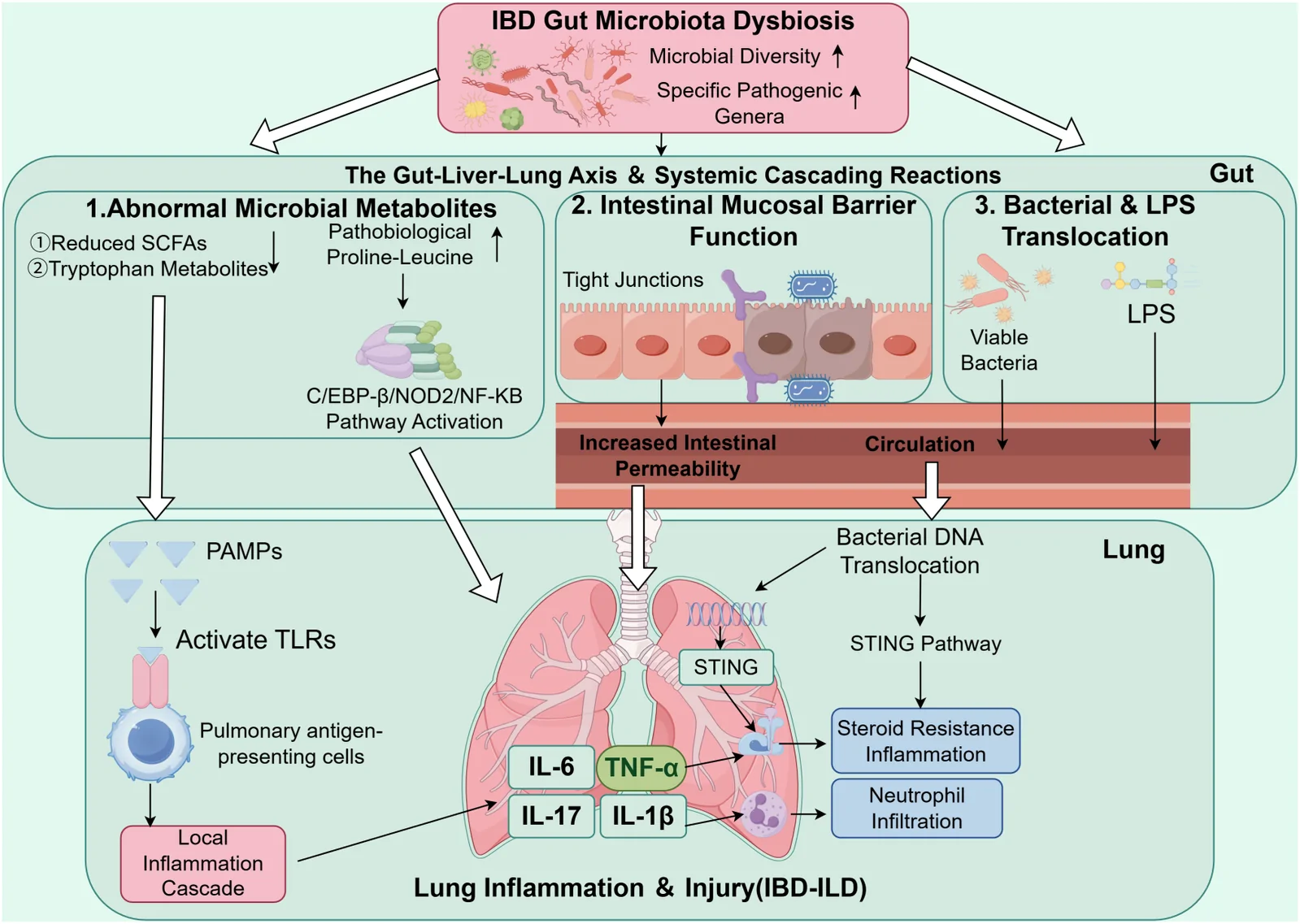
Schematic overview of the gut-lung axis-mediated pathogenic mechanisms in ibd-associated interstitial lung disease. This figure illustrates the three-tiered cascade linking gut microbiota dysbiosis to pulmonary inflammation in IBD-ILD. In the intestinal compartment, IBD is characterized by reduced microbial diversity, depletion of beneficial genera such as Muribaculaceae, and overgrowth of pathobionts. This dysbiosis leads to two interconnected downstream events: aberrant production of microbial metabolites, including decreased short-chain fatty acids (SCFAs) and tryptophan metabolites, alongside increased pathological metabolites such as proline-leucine and disruption of intestinal mucosal barrier integrity, characterized by downregulated tight junction proteins and increased permeability. At the systemic level, bacterial translocation permits the migration of live bacteria and microbial structural components (LPS) into circulation. Concurrently, metabolic imbalances remotely modulate pulmonary immune homeostasis via the bloodstream. In the pulmonary compartment, pathogen-associated molecular patterns (PAMPs) activate pattern recognition receptors (e.g., Toll-like receptors) on resident immune cells, triggering inflammatory cascades characterized by elevated IL-17, neutrophil infiltration, and upregulation of TNF-α, IL-6, and IL-1β. Bacterial DNA translocation further activates the STING pathway, promoting steroid-resistant inflammation. This gut-to-lung axis establishes a self-reinforcing cycle, as pulmonary inflammation reciprocally exacerbates gut dysbiosis and barrier dysfunction. Key molecules and pathways are indicated. SCFAs, short-chain fatty acids; LPS, lipopolysaccharide; PAMPs, pathogen-associated molecular patterns; TLRs, Toll-like receptors; Pro-Leu, proline-leucine.

### 3.1 Characteristics of gut microbiota dysbiosis in IBD patients and its association with lung injury

Gut microbiota dysbiosis in IBD is characterized by reduced microbial diversity and altered abundances of specific bacterial genera and is closely associated with the onset and progression of lung damage (Zhang et al., 2020; De Nuccio et al., 2022). In systemic sclerosis-associated ILD, patients exhibit distinct gut microbiota profiles, with specific bacterial species correlating with the extent of ILD on pulmonary imaging (Andréasson et al., 2026). Furthermore, studies using mouse models of pulmonary fibrosis have confirmed that the gut microbiota and its metabolites exhibit synchronous changes with fibrosis markers. The use of the gut microbiota to identify pulmonary fibrosis has demonstrated high accuracy, further supporting the hypothesis that gut microbiota dysbiosis may drive interstitial lung disease (Gong et al., 2021). Although the causal relationship between gastrointestinal dysbiosis and the development of ILD is not yet fully established, multiple studies have confirmed that alterations in the pulmonary microbiome and high microbial load are associated with poor prognosis and acute exacerbations in patients with connective tissue disease-associated interstitial lung disease (CTD-ILD) (Puppo and Carbone, 2026). It is worth noting that this association is bidirectional; pulmonary inflammation can, in turn, induce gut dysbiosis and intestinal barrier dysfunction, creating a vicious cycle (Popovic et al., 2023).

### 3.2 Long-distance regulation of pulmonary immune homeostasis by microbial metabolites

Metabolites produced by the gut microbiota are key mediators of the gut-lung axis’s long-distance immune regulatory effects, with short-chain fatty acids (SCFAs) and tryptophan metabolites playing central roles in maintaining pulmonary immune homeostasis (Zhang et al., 2024). SCFAs are produced by gut microbiota through the fermentation of dietary fiber. They reach the lungs via the bloodstream, where they activate G protein-coupled receptors (free fatty acid receptors) and inhibit histone deacetylases, thereby regulating pulmonary inflammatory responses and airway immune homeostasis (Ashique et al., 2022; Verma et al., 2024). Studies have shown that SCFAs not only directly influence pulmonary immune cells but also act on hematopoietic progenitor cells in the bone marrow, promoting the development of innate and adaptive immune cells and thereby alleviating airway inflammation (Dang and Marsland, 2019). In a mouse model colonized with carbapenem-resistant Enterobacteriaceae, the disappearance of the Muribaculaceae family in the gut led to reduced SCFA levels, which in turn caused a decrease in the number of alveolar macrophages and conventional dendritic cells, exacerbating the severity of pulmonary infection; however, SCFA supplementation or fecal microbiota transplantation restored the pulmonary immune response (Le Guern et al., 2023). In addition to SCFAs, tryptophan metabolites are important anti-inflammatory mediators; together with secondary bile acids, polyamines, and other compounds, they form a protective metabolic profile that inhibits fibroblast activation and collagen deposition, thereby reducing the severity of respiratory diseases (Beyoğlu and Idle, 2026). Conversely, pathological metabolites such as proline-leucine (Pro-Leu) can exacerbate pulmonary inflammation by activating the CCAAT/enhancer-binding protein beta (C/EBP-β)/nucleotide-binding oligomerization domain-containing protein 2 (NOD2)/nuclear factor kappa-B (NF-κB) signaling pathway (Yang et al., 2025). In UC-associated pulmonary injury models, elevated concentrations of linoleic acid and 7-hydroxy-3-oxocholic acid in bronchoalveolar lavage fluid have also been identified as potential metabolic biomarkers that link intestinal and pulmonary diseases (Tang et al., 2022). Imbalances in these metabolites are a key component of the pathogenesis of IBD-ILD (Mu et al., 2026).

### 3.3 The inflammatory cascade in the lungs induced by increased intestinal permeability and bacterial translocation

Bacterial translocation resulting from disruption of the intestinal mucosal barrier is a direct pathway through which the gut-lung axis mediates the inflammatory cascade in the lungs (Song et al., 2023; Mu et al., 2026). In a mouse model of DSS-induced colitis, disruption of the intestinal barrier was characterized by downregulation of tight junction protein expression and upregulation of permeability markers. Subsequent experiments using green fluorescent protein (GFP)-labeled fecal microbiota confirmed the migration of intestinal bacteria to the lungs, accompanied by elevated pulmonary IL-17 levels, neutrophil infiltration, and airway hyperresponsiveness (Eladham et al., 2025). This bacterial translocation involves not only the migration of live bacteria but also the systemic dissemination of microbial structural components, such as lipopolysaccharide (LPS). When pathogen-associated molecular patterns (PAMPs) produced by pathogenic bacteria enter the circulation, they can activate pattern recognition receptors (such as Toll-like receptors) on pulmonary antigen-presenting cells, triggering systemic low-grade inflammation and a lung-specific immune response (De Nuccio et al., 2022). In sepsis models, gut dysbiosis increases intestinal permeability, leading to the translocation of bacteria and their metabolites into the lungs, where they exacerbate lung injury by upregulating pro-inflammatory factors such as TNF-α, IL-6, and IL-1β (Yang et al., 2025). Furthermore, antibiotic-induced gut dysbiosis has been shown to increase intestinal permeability and disrupt the intracellular antimicrobial environment by altering the pulmonary transcriptome, thereby increasing susceptibility to pulmonary colonization (Han et al., 2024). Experiments with STING inhibitors have shown that blocking the STING pathway triggered by bacterial DNA translocation can effectively control colitis-associated pulmonary inflammation and restore steroid sensitivity, further validating the pathogenic role of bacterial translocation in the IBD-ILD inflammatory cascade (Eladham et al., 2026). This mechanism highlights the potential value of restoring the intestinal barrier and clearing exogenous microbial products in the treatment of IBD-ILD (Mu et al., 2026).

## 4 Microbiome-based therapeutic interventions

Based on the bidirectional regulatory mechanism of the gut-lung axis, microbiome modulators offer a new non-immunosuppressive adjunctive treatment option for IBD-ILD. Dysbiosis of the gut microbiota and alterations in its metabolites are key drivers of pulmonary inflammation and fibrosis. Restoring gut microbiota homeostasis through fecal microbiota transplantation (FMT), probiotics, or dietary interventions can alleviate lung injury (Wu et al., 2022; Yang et al., 2025; Thonghem et al., 2026). Animal studies have confirmed that FMT not only corrects gut microbiota dysbiosis but also significantly reduces the severity of bleomycin- or DSS-induced pulmonary inflammation and fibrosis. The underlying mechanisms involve restoring the integrity of the gut-lung barrier, upregulating the expression of tight junction proteins, and inhibiting the Toll-like receptor 4 (TLR4)/NF-κB signaling pathway (Jia et al., 2024; Ruan et al., 2024; Hu et al., 2026). Specific dietary components—such as soy isoflavones and Polygonatum polysaccharides—have also been shown to improve lung-gut barrier function by enriching bacterial genera that produce short-chain fatty acids (eg., *Akkermansia* and *Muribaculaceae*) and by regulating bile acid metabolism (Jia et al., 2024; Batu et al., 2026; Hu et al., 2026). Despite its promising prospects, the clinical translation of microbiome therapy for IBD-ILD still faces challenges, including donor standardization, immunological safety, and consistency of efficacy. Furthermore, most existing evidence comes from acute injury models, and high-quality clinical trial data targeting chronic fibrotic phenotypes remain lacking (Wu et al., 2026). Future research should focus on identifying specific combinations of “lung-protective” bacterial strains and metabolic effector molecules to develop standardized next-generation probiotic formulations.

## 5 Limitations, controversies, and unresolved issues in current research

Despite advances in gut-lung axis research, translating mechanistic insights into clinical applications for IBD-ILD remains a major challenge. Current studies predominantly focus on single organs or unidirectional pathways, overlooking the spatiotemporal dynamics of bidirectional gut-lung crosstalk. Animal models inadequately recapitulate the chronic, complex features of human IBD-ILD, and the causal role of microbiome alterations remains unconfirmed. Clinical trials are further challenged by endpoint selection that fails to capture the heterogeneous progression of both intestinal and pulmonary lesions. Addressing these limitations requires interdisciplinary collaboration and technological innovation.

### 5.1 The issue of fidelity in animal models simulating the pathological characteristics of the human gut-lung axis

Current animal models for IBD-ILD gut-lung axis research primarily rely on chemical inducers or gene-knockout mice. While these models have advanced our understanding of acute inflammatory cascades and microbial translocation, they poorly recapitulate the chronic, progressive nature and immunological complexity of human disease. Most models undergo repair or fibrosis following acute injury, failing to replicate the decades-long, relapsing course of human IBD-ILD (Wu et al., 2026). The bleomycin-induced fibrosis model, though useful for observing gut-lung microbiome-metabolite associations, is self-limiting and fundamentally differs from progressive human fibrosis (Hou et al., 2024; Wu et al., 2026). Additionally, genetic background profoundly influences phenotypic outcomes: only in susceptible contexts such as T-cell receptor delta (TCRδ) deficiency do DSS-induced colitis mice develop pulmonary changes, whereas wild-type mice do not, indicating that chemical induction alone cannot recapitulate human gene-environment-microbiome networks (Raftery et al., 2023). Finally, interspecies differences in microbiome composition, immune development, and mucosal barrier function necessitate validation of findings from mouse models in humans (Thonghem et al., 2026).

### 5.2 Establishing causal relationships: are microbial changes the cause or the effect

Whether gut microbiota dysbiosis drives pulmonary lesions, accompanies them, or results from them remains debated. Multiple observational studies confirm that IBD-ILD patients exhibit reduced gut microbial diversity and increased pathogenic bacteria, with specific microbial signatures linked to disease severity (Han et al., 2024; Hou et al., 2024; Thonghem et al., 2026), and Mendelian randomization has suggested nominal causal associations between the gut microbiota and extraintestinal manifestations (Lu et al., 2023). Conversely, reverse causality is equally plausible: pulmonary inflammation, hypoxia, and therapeutic drugs can all induce gut dysbiosis (Zhang et al., 2020; Ziaka and Exadaktylos, 2024; Liu et al., 2026). Studies on pneumoconiosis and acute lung injury models have demonstrated bidirectional lung-gut interactions, forming a self-reinforcing cycle that may be inflammation-driven rather than microbe-driven (Ziaka and Exadaktylos, 2024; Ma et al., 2025; Li et al., 2026). Intervention trials (FMT, probiotics) have shown inconsistent efficacy, which is confounded by disease activity, medications, and lifestyle factors (Liu et al., 2026; Wu et al., 2026). While antibiotic depletion or FMT has confirmed the mediating role of the gut microbiota in pulmonary fibrosis progression, large-scale longitudinal cohort studies in human IBD-ILD are still lacking to determine whether microbial changes preceded or followed pulmonary lesions (Ruan et al., 2024). Thus, without adequate control for confounders, microbial dysbiosis cannot be conclusively established as the cause of IBD-ILD.

## 6 Future research directions

Future research on IBD-ILD should prioritize three interconnected directions: multi-omics integration to identify robust biomarkers and decipher gut-lung crosstalk; standardized non-invasive diagnostic models based on stool, serum, or urine for early detection and real-time monitoring of pulmonary involvement; and comprehensive systems incorporating medication history, microbiome, metabolomics, and transcriptomics to distinguish drug-induced lung injury from primary disease, enabling precision diagnosis and personalized therapy.

### 6.1 The application of multi-omics technologies in identifying specific biomarkers of the gut-lung axis

Multi-omics integration has become a core approach for identifying IBD-ILD gut-lung axis biomarkers, as integrating metagenomic, metabolomic, transcriptomic, and proteomic data improves diagnostic accuracy and biological interpretability beyond single-omics analyses (Sauceda et al., 2022; Lee et al., 2026). Human Microbiome Project 2 demonstrated that combined metabolomic, viral, metagenomic, and metatranscriptomic scores achieved an AUC of 0.80 for diagnosing IBD, outperforming single-omics models (Arehart et al., 2024). For gut-lung axis-specific markers, elevated linoleic acid and 7-hydroxy-3-oxocholanolic acid in bronchoalveolar lavage fluid, along with concurrent changes in the pulmonary and gut microbiomes during UC-to-colorectal cancer progression, suggest that these metabolites and microbial communities are potential markers of intestinal-to-pulmonary disease spread (Tang et al., 2022). In IPF research, the integration of bulk RNA sequencing, single-cell transcriptomics, and peripheral blood multi-omics identified four core genes—C-X-C motif chemokine ligand 13 (CXCL13), interleukin-33 (IL33), Toll-like receptor 4 (TLR4), and insulin-like growth factor 1 (IGF1)—whose dysregulation was localized to fibroblasts and the immune microenvironment, with Mendelian randomization confirming causal associations with specific gut microbiota (Hu et al., 2026). Multi-view learning methods, such as StabilityCCA, have further identified combined biomarker structures associated with IBD disease states (Pusa and Rousu, 2024). Collectively, multi-omics technologies not only distinguish disease subtypes but also reveal shared pathogenic molecular signatures across the gut-lung axis.

### 6.2 Development of a non-invasive diagnostic model based on the microbiome and metabolome

Given the key mediating role of the gut-lung microbiomes and their metabolites, diagnostic models based on non-invasive samples (stool, serum, urine) have become a research hotspot. Metabolomics and virology show greater predictive value for IBD status than metagenomics and metatranscriptomics (Arehart et al., 2024). In COPD, the serum metabolite eremopetasinorol demonstrated high diagnostic accuracy, outperforming fecal markers and highlighting circulating metabolites as readouts of gut-lung axis regulation (Wang et al., 2026). In IBD, a cross-cohort analysis integrating nine metagenomic and four metabolomic cohorts identified three rare commensal bacteria (*Asaccharobacter celatus*) and 36 differential metabolites, achieving AUROC values of 0.92–0.98 across multiple global cohorts (Ning et al., 2023). A sparse neural encoder-decoder network integrating paired microbiome-metabolome data enabled high-accuracy prediction of IBD status and treatment response without diagnostic information, thereby revealing mechanisms via an interpretable microbiome-metabolome axis (Le et al., 2020). These non-invasive models provide practical tools for real-time monitoring of the gut-lung axis and early warning of pulmonary complications.

### 6.3 Strategies for distinguishing drug-induced lung injury from primary interstitial lung disease

Distinguishing drug-induced lung injury from primary IBD-ILD remains a clinical challenge that multi-omics technologies may address. These two entities differ in pathogenesis: drug-induced injury primarily involves drug metabolism and specific immune responses, whereas primary IBD-ILD is linked to dysregulation of the gut-lung axis, microbial translocation, and a shared genetic background. The stimulator of interferon genes (STING) pathway plays a key role in colitis-associated pulmonary inflammation; its inhibition controls inflammation and restores steroid sensitivity, suggesting that STING-related biomarkers may help differentiate infection- or inflammation-driven ILD from drug toxicity (Eladham et al., 2026). Metabolomics offers a new tool for differential diagnosis, as distinct etiologies may produce specific metabolic profiles (Marlicz et al., 2018). In acute lung injury treated with Tripterygium lactone, gut microbiota remodeling coupled with bile acid metabolic reprogramming represents a specific metabolic-microbial interaction pattern that may distinguish this entity from other lung injuries (Zha et al., 2025). Epigenetic markers, such as DNA methylation patterns, also hold potential to capture disease-related regulatory states; identifying drug-specific epigenetic alterations could help distinguish adverse drug reactions from natural disease progression (Lee et al., 2026). Future prospective studies should integrate medication history, microbiome, metabolomics, and host transcriptomics to establish comprehensive diagnostic models that improve accuracy.

## 7 Conclusion

IBD and ILD are interconnected through the gut-lung axis into a disease continuum. This review summarizes their molecular dialogue across epidemiological evidence, microbiota dysbiosis and metabolite-mediated immune regulation, bacterial translocation-triggered inflammation, and microbiome-based therapeutic prospects. Large-scale cohort studies confirm that IBD patients face a significantly elevated, likely causal risk of ILD. Mechanistically, gut dysbiosis reduces microbial diversity and alters metabolite profiles, modulating pulmonary immune homeostasis, while intestinal barrier disruption permits bacterial translocation, which activates pulmonary pattern recognition receptors and triggers inflammatory cascades, integrating immune cell trafficking, signaling convergence, and fibrotic remodeling.

Despite advances, challenges remain: animal models inadequately recapitulate human disease, the causality of microbiome alterations requires validation, and standards for subtype classification are lacking. Future research should prioritize multi-omics integration, non-invasive diagnostics, and systems that incorporate medication history, microbiome, metabolomics, and transcriptomics to distinguish drug-induced effects from primary disease, enabling precision diagnosis and personalized treatment. This review consolidates current knowledge, highlights gaps, and proposes directions to guide basic research and clinical translation.
